# Supplementary material for: Nucleophosmin supports WNT-driven hyperproliferation and tumor initiation
Source: Nat Genet. 2025 Dec 18;58(1):100–15. doi: 10.1038/s41588-025-02408-7 (PMC12807877; doi:10.1038/s41588-025-02408-7)
Supplement: Supplementary file 1 — Supplementary Note. [file 41588_2025_2408_MOESM1_ESM.pdf]

nature genetics

Supplementary information https://doi.org/10.1038/s41588-025-02408-7

Nucleophosmin supports WNT-driven hyperproliferation and tumor initiation

In the format provided by the authors and unedited

Supplementary Note – Additional Methods

Histology and immunohistochemistry

Tissues were fixed in 10% neutral buffered formalin overnight in either room temperature or at 4 ^{o}C and subsequently embedded in paraffin wax. For BrdU scoring in short term experiments intestinal tissue was fixed in a solution of methanol, chloroform and acetic acid, mixed in a 4:2:1 ratio, for 24 h before being transferred to formalin and paraffin embedded (FFPE). Immunohistochemistry (IHC) and *in situ* hybridisation (ISH) was performed on 4 µm FFPE sections which had previously been heated at 60 °C for 2 h.

The following antibodies were stained on a Leica Bond Rx autostainer at an optimal dilution: Lysozyme (1:200, Agilent, A099), p21 (1:250, Abcam, ab107099), phospho-4E-BP1 (1:250, Cell Signaling Technology (CST), 2855), phospho-eEF2 (1:100, CST, 2331) and phospho-eIF2α (1:50, CST, 3398), phospho-eIF4e (1:150, Abcam, ab76256), phospho-histone H2A.X (20E3) (1:120, CST, 9718). All FFPE sections underwent on-board dewaxing (Leica, AR9222) and epitope retrieval for lysozyme staining was performed using enzyme 1 solution (Leica, AR9551) for 5 min at 37°C, for p21, p-4E-BP1, p-eEF2, p-eIF2α and phospho-H2A.X staining with ER2 solution (Leica, AR9640) for 20 min at 95°C, while for p-eIF4E with ER1 solution (Leica, AR9661) for 40min. Sections were rinsed with Leica wash buffer (Leica, AR9590) before peroxidase block was performed using an Intense R kit (Leica, DS9263) for 5 min, and rinsed with wash buffer before sections for p21 staining had blocking solution applied from the Rat ImmPRESS kit (Vector Labs, MP7444-15) for 20 min. Sections were rinsed with wash buffer before primary antibody application, and then rinsed again. Sections being stained for Lysozyme, p4-EBP1, p-eEF2, p-eIF2α, p-eIF4E and phospho-H2A.X had rabbit envision (Agilent, K4003) applied and sections for p21 staining had rat ImmPRESS secondary antibody applied for 30 min before rinsed with wash buffer, visualised using DAB (Agilent, K3468), and counterstained with haematoxylin in the Intense R kit. For β-catenin (1:50, BD Biosciences, 610154) staining, tissue was fixed at 4 °C for no more than 24h before immunostaining.

FFPE sections for BrdU (1:250, BD Biosciences, 347580), cleaved PARP (E51) (1:1000, Abcam, ab32064), Glutamine Synthetase (1:800, Sigma-Aldrich, HPA-007316), Ki67 Clone MIB-1 (1:100, Agilent, M7240), NPM1 (1:50, CST, 3542), and p53 (clone CM5) (1:750, Leica, NCL-L-p53CM5p) staining were loaded into an Agilent pre-treatment module to be dewaxed and undergo heat induced epitope retrieval (HIER) using an appropriate target retrieval solution (TRS) where sections were heated to 97°C for 20 min. Sections for BrdU, cleaved PARP, Ki67, NPM1 and p53 staining underwent epitope retrieval using High pH TRS (K8004, Agilent) and sections for GS were retrieved using Low pH TRS (Agilent, K8005). After HIER the sections were rinsed in flex wash buffer (Agilent, K8007) prior to being loaded onto the Agilent autostainer. The sections underwent peroxidase blocking (Agilent, S2023) for 5 min and rinsed with flex buffer. Sections for BrdU antibody staining had mouse Ig block (Vector Labs, MKB-2213) applied for 20 min then rinsed with flex buffer. Primary antibody application was followed by section washing with flex buffer before

application of the appropriate envision secondary antibody (Agilent). Sections for BrdU and Ki67 staining had mouse envision (K4001) and sections for GS, cleaved PARP, NPM1 and p53 had rabbit envision (K4003) applied for 30 min. Sections were rinsed with flex wash buffer before applying Liquid DAB (Agilent, K3468) for 10 min. Sections were washed in water and counterstained with haematoxylin z (CellPath, RBA-4201-00A).

ISH detection for *47S rRNA* (Bio-Techne, 417348), *Olfm4* (Bio-Techne, 311838), *Ppib* (Mm-PPIB positive control, Bio-Techne, 313918) and *Dlgap1* (dapβ negative control, Bio-Techne, 312038) mRNA was performed using RNAScope 2.5 LSx (Brown) detection kit (322700; Bio-Techne) according to the manufacturer's instructions.

Staining for AB/PAS was performed manually on FFPE sections that were dewaxed and rehydrated through xylene and a graded ethanol series before washing in water. Rehydrated slides were stained for 10 min in Alcian blue solution before rinsing in tap water. Sections were placed in 0.5% periodic acid (Leica, 3803812) for 7 min before washing in tap water and transferred to Schiff's reagent (CellPath, H5265-500) for 20 min. The sections were washed in tap water to terminate the reaction.

To complete the IHC, ISH and AB/PAS staining, sections were rinsed in tap water, dehydrated through graded ethanols and placed in xylene. The stained sections were coverslipped in xylene using DPX mountant (CellPath, SEA-1300-00A).

BrdU and p21 scoring in the intestine was performed by manual counting of positively stained cells in ≥25 half crypts per mouse and then calculating the average values. Quantification of p53+ cells, p21+ hepatocytes, *47S rRNA* and *Olfm4* ISH, as well as H-score values for IHC stained samples, were all performed using HALO Image Analysis Platform (v3.6.4134, Indica Labs, Inc.) trained to detect intestinal epithelial cells or hepatocytes and quantify the optical density of cellular staining.

Immunofluorescence and digital image analysis

Immunofluorescence staining for fibrillarin (1:50, CST, 2639) and p53 (1:500, Leica, NCL-L-p53CM5p) was performed on a Leica Bond Rx autostainer on sections prepared as described above. Subsequently, FFPE sections underwent on-board dewaxing (Leica, AR9222) and epitope retrieval using ER2 retrieval solution (Leica, AR9640) for 20 minutes at 95 °C. Sections were rinsed with Leica wash buffer (Leica, AR9590) before blocking using normal goat serum (Vector Labs, S1012) for 20 minutes. Sections were rinsed with wash buffer, primary antibody was applied and then rinsed again with wash buffer before goat anti-rabbit AF647 (1:250, Invitrogen, A21244) was applied for 30 minutes. The sections were then rinsed with wash buffer and DAPI solution (Sigma-Aldrich, MDB0015) was applied. Sections were finally rinsed in deionised water prior to coverslip attachment using ProLong diamond mountant (Thermo Scientific, P36970).

Fluorescently stained slides were scanned on the Evident VS200 (version ASW 4.1.1). Brightfield slide overview scans were scanned at 4x magnification (1.625

μm/pixel) and subsequently scanned with the Maximum Intensity Projection (MIP) scan mode at 60x (oil) magnification (0.1083 μm/pixel) using the X-Cite NOVEM light source. Manual exposure times for CY5 and DAPI filter cubes were set based on the whole scan project at 89.998ms and 1.137ms, respectfully.

Image analysis for fibrillarin took place in Visiopharm (version 2024.07.2.17212 x64) on mouse SI. Crypt, villi, and other tissue regions were manually annotated with their corresponding regions of interest (ROIs) layers to prepare for single cell and fibrillarin spot analysis. For nuclear detection, a new analysis protocol package (APP) was created using the deep learning module with U-Net. Mouse SI images were used as the training substrate. These images were manually annotated to assign labels to nuclei, nuclear boundaries, and background. The APP was then trained with approximately 94,000 iterations using the DAPI channel as an input, generating corresponding nuclei, boundary, and background feature probability heatmaps. The nuclei feature was then thresholded to assign nuclear labels to pixels with 60% or above confidence. Nuclei were then outlined with an 'annotation layer' in post-processing steps to prepare for fibrillarin spot detection. For spot detection, an APP was developed using the deep learning module with U-Net to label fibrillarin spots within nuclear annotations. Mouse SI images were used as the training substrate. These images were manually annotated to assign labels to fibrillarin spots and background. The APP was then trained with over 100,000 iterations with CY5 (fibrillarin channel) as the input to generate fibrillarin and background feature probability heatmaps. The fibrillarin feature was then thresholded to assign labels to pixels with 60% or above confidence. XY coordinates of nuclei and spots were calculated. Mean pixel intensity measures of fibrillarin were calculated for each spot and nuclear annotation, as well as roundness and area of each spot being calculated in the entire image and within each crypt and in villi region of interest. Data was then exported for subsequent downstream analysis for each genotype.

p53-positive cells within the crypts of the small intestinal epithelium were quantified using QuPath. Crypts were annotated using the brush tool and cells detected by DAPI staining using the QuPath cell detection function. Next, a classifier was determined for both DAPI and p53 (i.e. fluorophore AFA). The object classifier was loaded and the number of DAPI positive, and DAPI+p53 positive cells determined. Quality control was performed to remove any non-epithelial cells and artefacts from the quantification. The total number of DAPI-positive and DAPI-positive p53-positive cells was then retrieved for downstream analysis.

Epithelial extract preparation for omics approaches

Crypt fractions were isolated from proximal small intestine in a two-step process. Firstly, the intestine was flushed with PBS, cut lengthwise, and incubated for 7 min at 37 °C with regular agitation in RPMI 1640 medium (Thermo Fisher Scientific, 21875059) supplemented with 10 mmol/L EDTA and 200 μg/mL cycloheximide (Sigma-Aldrich, C7698). Next, the tissue was transferred to ice-cold PBS containing 10 mmol/L EDTA and 200 μg/mL cycloheximide and incubated on ice for 7 min with regular agitation. Finally, the tissue was removed and the PBS fraction spun at 350 g

at 4 ^{o}C to isolate crypts, which were snap frozen in liquid nitrogen, and kept at -80 ^{o}C prior to further processing. Samples from each animal were divided and independently processed for Riboseq and proteomic analysis.

RNA extraction and qPCR

Murine tissues were homogenised in Precellys CK14 tubes and RNA was isolated by RNAeasy kits (QIAGEN, 74104) following the manufacturer's protocol with on-column DNAse digestion. RNA concentrations were determined with a NanoDrop 200c spectrophotometer (Thermo Scientific). cDNA was synthesised using the high capacity cDNA reverse transcription kit (Applied Biosystems, 10704217) and random hexamers. qPCRs were performed using the DyNAmo HS SYBR Green qPCR Kit (Thermo Scientific, F410L) on a Bio-Rad C1000 Touch CFX96 real-time system using the CFX Maestro 2.3 software. Primer sets used for qPCRs are provided in Supplementary Table 1.

Cell culture

Primary organoid lines were generated from the small intestines of biologically independent mice. They were maintained in 5% CO_2 37 ^{o}C in Matrigel (Corning, 356231) and Advanced DMEM/F12 (Life Technologies, 12634-028) cell culture medium supplemented with 2 mmol/L l-glutamine (Life Technologies, 25030-024), 5 mmol/L HEPES (Life Technologies, 15630-080), 100 U/mL penicillin/streptomycin (Life Technologies, 1540-122), 1X N2 (Invitrogen, 17502-048), 1X B27 (Invitrogen, 12587-010), 100 ng/mL noggin (PeproTech, 250-38), 50 ng/mL EGF (PeproTech, AF-100–15) and 500 ng/mL R-spondin (R&D Systems, 3474-RS). For *in vitro* inductions 4-OH tamoxifen (Sigma, H7904) was used at 1 µM. Tunicamycin (Sigma-Aldrich, T7765) was used at final concentration of 25 µg/ml in cell culture medium for 5 h. Treatment with 1 µM ISRIB (Sigma-Aldrich, SML0843) was performed for 24 h. For puromycin incorporation assays, media was replaced with fresh 4 h prior to harvest and puromycin (Invivogen, ant-pr) was added at 10 µg/ml final concentration for the last 30 min of drug treatments. As negative control, treatment with 100 µg/ml cycloheximide (Sigma-Aldrich, C7698) was also performed for the final 30 min before cell harvesting.

^{35}S-methionine labelling

Organoids were examined at peak growth stage; specifically, three days post-passaging and six hours following media refresh. To assess protein synthesis, ^{35}S-methionine (PerkinElmer, NEG772002MC) was added at a concentration of 30 µCi/mL for 30 min prior to cell harvesting and lysis using RIPA lysis buffer. Proteins were precipitated with 12.5% (w/v) trichloroacetic acid onto glass microfiber filters (Whatman, 1827-024) via a vacuum manifold, followed by sequential washes with 70% ethanol and acetone. Radioactive signal was quantified using a Wallac MicroBeta

TriLux 1450 scintillation counter with MicroBeta Workstation software (Version 4.0) (PerkinElmer) and Ecoscint liquid scintillation cocktail (SLS, LS271). Readings were normalized to total protein levels measured using the BCA protein assay. The rate of protein synthesis was calculated as counts per minute per microgram of protein (CPM/µg protein), relative to a control group.

Western blotting

Organoids were collected by centrifugation (1000 g for 5 min), lysed in RIPA lysis buffer and lysates were cleared by centrifugation (16000 g, 10 min, 4 °C). Protein samples were resolved on 4-12 % denaturing polyacrylamide gels (Invitrogen, WXP41220BOX) and were transferred onto 0.2 µm nitrocellulose membranes (Amersham). After blocking, blots were probed overnight with ATF4 (1:1000, CST, 11815), BiP (1:5000, Abcam, ab21685), CHOP (1:1000, CST, 2895), eIF2α (1:1000, CST, 9722), phospho-eIF2α, (1:500, Abcam, ab32157), GADD34 (1:1000, Proteintech, 10449-1-AP), NPM1 (1:1000, CST, 3542), p21 (1:1000, CST, 64016), p53 (1:1000, Abcam, ab26) and vinculin (1:10000, Abcam, ab129002) antibodies. After incubation with Goat anti-mouse IgG HRP (1:10000, Invitrogen, A16078) or Goat anti-rabbit IgG HRP (1:10000, Invitrogen, A16110) secondary antibodies, the blots were developed using the ECL system (Thermo Scientific SuperSignal West Pico PLUS chemiluminescent substrate, 34577, or Bio-Rad Clarity Max Western ECL Substrate, 170562). For fluorescent western blotting for puromycin (1:10000, Sigma, MABE343), NPM1 (used as above) and β-actin (1:5000, Sigma, A2228) the secondary antibodies Alexa Fluor™Plus 800 (1:10000, Thermo Scientific, A32735) and Alexa Fluor™ 680 (1:10000, Thermo Scientific, A21057) were used and visualised and quantified using LI-COR Odyssey CLx with Image Studio v6.0 (1.0.22) and Empiria Studio Software v3.0.0.173.
