# Supplementary material for: Nucleophosmin supports WNT-driven hyperproliferation and tumor initiation
Source: Nat Genet. 2025 Dec 18;58(1):100–15. doi: 10.1038/s41588-025-02408-7 (PMC12807877; doi:10.1038/s41588-025-02408-7)
Supplement: Supplementary file 2 — Reporting Summary [file 41588_2025_2408_MOESM2_ESM.pdf]

nature portfolio

Corresponding author(s): Georgios Kanellos
Owen Sansom

Last updated by author(s): Sep 29, 2025

# Reporting Summary

Nature Portfolio wishes to improve the reproducibility of the work that we publish. This form provides structure for consistency and transparency in reporting. For further information on Nature Portfolio policies, see our Editorial Policies and the Editorial Policy Checklist.

## Statistics

For all statistical analyses, confirm that the following items are present in the figure legend, table legend, main text, or Methods section.

| n/a | Confirmed | |
|---|---|---|
| ☐ | ☒ | The exact sample size (*n*) for each experimental group/condition, given as a discrete number and unit of measurement |
| ☐ | ☒ | A statement on whether measurements were taken from distinct samples or whether the same sample was measured repeatedly |
| ☐ | ☒ | The statistical test(s) used AND whether they are one- or two-sided<br>*Only common tests should be described solely by name; describe more complex techniques in the Methods section.* |
| ☒ | ☐ | A description of all covariates tested |
| ☐ | ☒ | A description of any assumptions or corrections, such as tests of normality and adjustment for multiple comparisons |
| ☐ | ☒ | A full description of the statistical parameters including central tendency (e.g. means) or other basic estimates (e.g. regression coefficient) AND variation (e.g. standard deviation) or associated estimates of uncertainty (e.g. confidence intervals) |
| ☐ | ☒ | For null hypothesis testing, the test statistic (e.g. *F*, *t*, *r*) with confidence intervals, effect sizes, degrees of freedom and *P* value noted<br>*Give P values as exact values whenever suitable.* |
| ☒ | ☐ | For Bayesian analysis, information on the choice of priors and Markov chain Monte Carlo settings |
| ☒ | ☐ | For hierarchical and complex designs, identification of the appropriate level for tests and full reporting of outcomes |
| ☐ | ☒ | Estimates of effect sizes (e.g. Cohen's *d*, Pearson's *r*), indicating how they were calculated |

*Our web collection on statistics for biologists contains articles on many of the points above.*

## Software and code

Policy information about availability of computer code

| | |
|---|---|
| Data collection | ZEN 2 (blue edition, v2.0.0.0) (Carl Zeiss Microscopy)<br>Wallac MicroBeta TriLux 1450 scintillation counter with MicroBeta Workstation software (Version 4.0) (PerkinElmer)<br>Evident VS200 (version ASW 4.1.1) for fluorescence slide scanning<br>LI-COR - Odyssey CLx with Image Studio v6.0 (1.0.22) Image Studio (LICORbio)<br>BIO-RAD ChemiDoc Imaging System (Image Lab Software version 2.3.0.07)<br>Xcalibur software (Thermo Scientific, version 4.1.31.9)<br>Bio-Rad C1000 Touch CFX96 real time PCR system with CFX Maestro 2.3 Software (5.3.022.1030) |
| Data analysis | Microsoft Excel 2016 (16.0.5487.1000)<br>Prism version 7.0.4 (GraphPad)<br>HALO Image Analysis Platform version 3.6.4134 (Indica Labs, Inc.)<br>ImageJ (version 1.53q)<br>R (versions 4.2.2, 4.3.2 and 4.3.3)<br>RStudio (version 2023.12.1 Build 402) (Posit)<br>Image Studio v6.0 (1.0.22) (LICORbio)<br>Empiria Studio Software v3.0.0.173 (LICORbio)<br>Aperio ImageScope Version 12.4.3.5008 (Leica)<br>SPSS version 28.0.0 (IBM)<br>Kaplan-Meier Plotter (online tool)<br>Survminer package in RStudio version 3.0.3<br>Counts from TCGA data were normalised using the VST function from DESeq2 (version 1.36) |

ggplot2 (v.3.5.1)
ggpubr (v.0.6.0)
ggbeeswarm (v.0.7.2)
FastQC version 0.11.8
Trim Galore version 0.6.4
HISAT2 version 2.1.0
FeatureCounts version 1.6.4
DESeq2 version 1.22.2
ReactomePA version 1.36.0.
Visiopharm version 2024.07.2.17212 x64
QuPath Version: 0.5.1
gsva function from the Python package GSEApy (v1.1.8)
For copy number (CN) status, we used the absolute CN values calculated by GISTIC2 (v2.0.23)
WGCNA (v.1.70-3) R package
PDSclassifier (v.1.0.0) R package
ProliferativeIndex (v.1.0.1) R package
The gene signature DNA repair (Hallmark) was accessed via msigdbr (v.7.4.1) R package to obtain single sample gene set enrichment analysis (ssGSEA) score using GSVA (v.1.42.0) R package.
MS Raw data were processed with MaxQuant software version 1.6.14.0 and searched with Andromeda search engine.
MaxQuant output was used for protein quantitation analysis using Perseus software version 1.6.15.0.
Riboseq analysis was performed following the publicly available pipeline from the Bushell lab (https://github.com/Bushell-lab/Ribo-seq). Shell scripts analyses were run on a terminal in Ubuntu 20.04.6 LTS; R scripts were run in RStudio 2023.12.0, Build 369 using R versions 4.2.2, 4.3.2 (2023-10-31), and 4.3.3. The code used specifically in this project for Riboseq analysis and integration with the proteomics data is available on the project-specific GitHub page (https://github.com/ChiaraGiacomelli/NPM_CRC).

For manuscripts utilizing custom algorithms or software that are central to the research but not yet described in published literature, software must be made available to editors and reviewers. We strongly encourage code deposition in a community repository (e.g. GitHub). See the Nature Portfolio guidelines for submitting code & software for further information.

## Data

Policy information about availability of data

All manuscripts must include a data availability statement. This statement should provide the following information, where applicable:

- Accession codes, unique identifiers, or web links for publicly available datasets
- A description of any restrictions on data availability
- For clinical datasets or third party data, please ensure that the statement adheres to our policy

RNA sequencing data generated in this study have been deposited in the Gene Expression Omnibus (GEO) database under the accession numbers GSE230110, GSE309379 and GSE250047. Ribosome profiling data have been deposited at GEO under the accession number GSE249958. Proteomics data have been deposited to the ProteomeXchange Consortium via the PRIDE partner repository with the dataset identifier PXD062969. Murine liver Npm1 expression data presented in Extended Data Fig. 10a were derived from RNAseq data available at GEO under accession number GSE230137. Human cancer analyses were conducted using data that are in whole or part based upon data generated by the TCGA Research Network (https://www.cancer.gov/tcga), as well as the publically available colon cancer microarray dataset GSE39582. Source data are available with this manuscript.

## Research involving human participants, their data, or biological material

Policy information about studies with human participants or human data. See also policy information about sex, gender (identity/presentation), and sexual orientation and race, ethnicity and racism.

| | |
|---|---|
| Reporting on sex and gender | CRC PATIENT COHORT ASSESSED FOR NPM1 EXPRESSION LEVELS<br>Sex was assigned based on medical records and was not considered in the present study. |
| Reporting on race, ethnicity, or other socially relevant groupings | No socially constructed or socially relevant categorization variables were used in this study. |
| Population characteristics | NPM1 protein expression was assessed in a retrospectively collected cohort of stage I-III CRC patients (n=787). The cohort consisted of CRC patients undergoing surgical resection with curative intent within Greater Glasgow and Clyde National Health Service between 1997 and 2013. Data are deposited within the Glasgow Safehaven (GSH21ON009).<br>The age of the patients included in this cohort ranged from 21-98 years with a mean of 68.79 years. The cohort consisted of 45%/55% female/male patients. |
| Recruitment | This was a retrospectively collected cohort. Patients were excluded from the study due to mortality within 30 days of surgery, administration of neoadjuvant therapy and/or emergency presentation. |
| Ethics oversight | Ethical approval was in place for the study (MREC/01/0/3). |

Note that full information on the approval of the study protocol must also be provided in the manuscript.

# Field-specific reporting

Please select the one below that is the best fit for your research. If you are not sure, read the appropriate sections before making your selection.

☒ Life sciences ☐ Behavioural & social sciences ☐ Ecological, evolutionary & environmental sciences

For a reference copy of the document with all sections, see nature.com/documents/nr-reporting-summary-flat.pdf

# Life sciences study design

All studies must disclose on these points even when the disclosure is negative.

| | |
|---|---|
| Sample size | No formal power analyses were carried out for determining cohort sizes in the present study. All in vivo cohort sizes were determined based on power analyses in studies previously carried out in our lab in similar GEM models, respecting the limited use of animals in line with the 3R system: Replacement, Reduction, Refinement.<br>The human colorectal cancer cohort analysed for NPM1 expression as part of this study was based on historic patient samples and no prospective sample collection carried out as part of this study.<br>For in vitro experiments the minimum number of biological replicates required was used that permitted meaningful statistical comparisons. |
| Data exclusions | Patients represented on the human CRC TMA were excluded due to mortality within 30 days of surgery and/or administration of neoadjuvant therapy.<br>As part of QC in the Riboseq analysis, any transcript with average reads across all samples lower than 10 was excluded from any downstream analysis.<br>No other data were excluded from the analyses in the present study. |
| Replication | All in vivo experiments are performed in individual, biologically unique, animals. As such, all replicate values in all experiments presented correspond to independent replicate animals that confirm reproducibility of the effect of genetic manipulation and/or drug treatments on these animals. All numbers of individual biological replicates are stated for every experiment and denoted with 'n'.<br><br>35S methionine incorporation in organoids in vitro was performed in 3 independent organoid lines, derived from 3 independent animals (3 technical replicates each) |
| Randomization | To minimise genetic variability, all experimental and control animals were generated on inbred genetic backgrounds. Where possible, control and experimental animals were co-housed independent of genotype. All aging experiments included animals from both sexes. Animals were recruited to treatment groups in a partially randomised manner while taking these factors into account.<br><br>In vitro organoid experiments were performed on a single defined genetic background. As such, no randomization was performed. |
| Blinding | Researchers were not blinded to genotype during study and data collection. The viability of NPM1 knockout in adult organisms had not been previously assessed and, as such, the knockout animals needed close monitoring. In addition, the preclinical cancer models used require enhanced inspection to ensure animal welfare and compliance with Home Office regulations. The researchers were blinded to genotype and/or drug treatment during data analyses. |

# Reporting for specific materials, systems and methods

We require information from authors about some types of materials, experimental systems and methods used in many studies. Here, indicate whether each material, system or method listed is relevant to your study. If you are not sure if a list item applies to your research, read the appropriate section before selecting a response.

## Materials & experimental systems

| n/a | Involved in the study | |
|---|---|---|
| ☐ | ☒ | Antibodies |
| ☐ | ☒ | Eukaryotic cell lines |
| ☒ | ☐ | Palaeontology and archaeology |
| ☐ | ☒ | Animals and other organisms |
| ☒ | ☐ | Clinical data |
| ☒ | ☐ | Dual use research of concern |
| ☒ | ☐ | Plants |

## Methods

| n/a | Involved in the study | |
|---|---|---|
| ☒ | ☐ | ChIP-seq |
| ☒ | ☐ | Flow cytometry |
| ☒ | ☐ | MRI-based neuroimaging |

## Antibodies

| | |
|---|---|
| Antibodies used | Antibody - Company - Code - (Lot Number) - Dilution<br>Brdu - BD - Biosciences - 347580 - (3016583) - 1:250 (IHC)<br>Glutamine Synthetase - Sigma-aldrich - HPA007316 - (000016878) - 1:800 (IHC)<br>Lysozyme - Agilent - A099 - (41480045) - 1:200 (IHC)<br>NPM1 - Cell Signaling - 3542 - (5) - 1:50 (IHC); 1:1000 (WB) |

p21 - Abcam - ab107099 - (1029985-2) - 1:250 (IHC)
p53 (clone CM5) - Leica Biosystems - NCL-L-p53CM5p - (6087005) - 1:750 (IHC); 1:500 (IF-IHC)
cleaved PARP [E51] - Abcam - ab32064 - (1091190-1) - 1:1000 (IHC)
Phospho-Histone H2A.X (Ser139) (20E3) - CST - 9718 - (21) - 1:120 (IHC)
phospho-4E-BP1 - Cell Signaling - 2855 - (26) - 1:250 (IHC)
phospho-eEF2 - Cell Signaling - 2331 - (9) - 1:100 (IHC)
phospho-eIF2α - Cell Signaling - 3398 - (8) - 1:50 (IHC); 1:500 (WB)
phospho-eIF4e - Abcam - ab76256 - (1008447-6) - 1:150 (IHC)
β-catenin - BD Biosciences - 610154 - 1:50 (IHC)
Fibrillarin - CST - 2639 - (4) - 1:50 (IF-IHC)
Ki67 Clone MIB-1- Agilent - M7240 - (41584781) - 1:100 (IHC)
Mouse Envision - Agilent - K4001 - (11605675)
Rabbit Envision - Agilent - K4003 - (11623090)
Rat ImmPRESS kit - Vector Labs - MP-7444-15 - (ZK0118)
eIF2α - Cell Signaling - 9722 - (9) - 1:1000 (WB)
Vinculin - Abcam - ab129002 - 1:10000 (WB)
BiP - Abcam - ab21685 - (10) - 1:5000 (WB)
ATF4 - CST - 11815) - (6) - 1:1000 (WB)
GADD34 - Proteintech 10449-1-AP - (00102093) - 1:1000 (WB)
CHOP - CST - 2895 - (15) - 1:1000 (WB)
p53 [PAb 240] - Abcam - ab26 - 1:1000 (WB)
p21 - CST - 64016 - 1:1000 (WB)
puromycin - Sigma - MABE343 - (3305878) - 1:10000 (WB)
Beta-actin - Sigma - A2228 - (052M4816V) - 1:5000 (WB)
Goat anti-mouse IgG HRP - Invitrogen - A16078 (98-56-050423) - 1:10000 (WB)
Goat anti-rabbit IgG HRP - Invitrogen - A16110 - 1:10000 (WB)
Goat anti-Rabbit IgG (H+L) Alexa Fluor™Plus 800 - Thermo Scientific - A32735 - (ZB382373) - 1:10000 (WB)
Goat anti-Mouse IgG (H+L) Cross-Adsorbed Secondary Antibody, Alexa Fluor™ 680 - Thermo Scientific - A21057 - (2795341) - 1:10000 (WB)

Validation

All antibodies were used according to the manufacturers' instructions.

Anti-BrdU antibody, clone B44, is derived from hybridization of Sp2/0-Ag14 mouse myeloma cells with spleen cells from BALB/c mice immunized with iodouridine-conjugated ovalbumin. The antibody also reacts with iodouridine. Tissues derived from mice with no prior administration of BrdU have been stained as a negative control.

Glutamine Synthetase (Anti-GLUL) antibody produced in rabbit, is developed and validated by the Human Protein Atlas (HPA) project. Each antibody is tested by immunohistochemistry against hundreds of normal and disease tissues. These images can be viewed on the Human Protein Atlas (HPA) site. The antibodies are also tested using immunofluorescence and western blotting.

Lysozyme antibody staining on murine tissue has been verified in our recent paper in nature communications with DOI: 10.1038/s41467-023-44342-4, where oncogenic KRAS signalling suppresses Paneth cell differentiation.

NPM1 antibody has been validated within this study by comparing NPM1 proficient to NPM1 knockout tissue.

p21 antibody has been validated by the company by analysis of mouse p21WT fibrosarcoma and mouse p21KO fibrosarcoma tissue sections labeling p21 with ab107099.

p53 NCL-L-p53-CM5p is specific for mouse and rat p53 protein according to the manufacturer. It has also been validated within the CRUK Scotland Institute after staining murine sections following MDM2 knockout.

cleaved PARP antibody has been knock-out validated by the company were it was shown to react with Cleaved PARP in wild-type HAP1 cells treated with 1uM staurosporine for 3hrs in Western blot, with loss of signal observed in PARP knockout sample.

phospho-histone H2A.X antibody has been validated by Western blot analysis of extracts from untreated or UV-treated 293 cells by the company. It has also been validated to react with various cancerous tissues by IHC, and we also show increased reactivity to NPM1 KO murine intestinal tissue with lack of signal in corresponding control tissue in Extended Data Figure 8n.

phospho-4E-BP1 monoclonal antibody is produced by immunizing animals with a synthetic phosphopeptide corresponding to residues surrounding Thr37 and Thr46 of mouse 4E-BP1. It has been validated by the company to be reactive to murine tissue.

phospho-eEF2 has been verified for use in tissue sections in our lab and published material can be found in this published article doi: 10.7554/eLife.69729.

phospho-eIF2α antibody specificity has been confirmed by the company by treating cells with thapsigargin, which induces unfolded protein response, and confirming induction of phospho-eIF2α expression. Furthermore, In our manuscript we confirm the staining is absent when we treat with inhibitor against PERK, a kinase responsible for eIF2α phosphorylation.

phospho-eIF4e antibody has been validated by the company as well as in our lab in MNK1/2 knockout murine tissue samples (https://doi.org/10.1158/2159-8290.CD-20-0652).

For the β-catenin antibody we provide validation and specificity within our own data by showing nuclear translocation post APC loss in multiple murine tissues (see Extended Data Figure 1b).

Fibrillarin antibody specificity has been validated by CST for Western blot (WB) and immunofluorescence (IF/ICC) across multiple species (human, mouse, rat, and monkey), and it detects endogenous fibrillarin. Many peer-reviewed publications cite use of this

antibody and within our study it reliably localised to nucleoli.

Anti-Ki67 antibody is well established for immunohistochemichal use and cited widely. Some examples that confirm reactivity and reduced signal post drug treatments or genetic manipulations include doi.org/10.1038/s41388-019-1047-4, doi/10.1126/sciadv.abi7511 and doi.org/10.1038/s41467-019-08839-1.

eIF2α antibody has been assessed by western blot analysis of extracts from PC12 cells, untreated or thapsigargin-treated.

Vinculin antibody specificity confirmed with VCL knockout cell line validation by the company.

BiP antibody is validated for use in ICC/IF, IHC-P, WB in human, mouse, recombinant fragment samples by the company and has been cited over 646 times in peer reviewed journals.

ATF4 antibody has been validated by western blot analysis of extracts from 293 and HeLa cells, untreated (-) or tunicamycin-treated by the company, as well as by us using unicamycin-treated murine intestinal organoids.

GADD34 has been validated by WB anaysis of si-Control and si-GADD34 transfected PC-3 cells by the company.

CHOP antibody has subjected to western blot analysis of extracts from C6 and A-204 cells, untreated or treated with thapsigargin (300 nM, 2 hours) or tunicamycin (24 µg/ml, 2 hours) to promote stress response.

p53 [PAb 240] antibody has been KO validated for confirmed specificity by the company. ab26 was shown to specifically react with p53 in wild type HCT116 cells treated with irinotecan. No band was observed in p53 knockout HCT116 cells.

p21 (CST - 64016) antibody has been validated for Western blot by the company by analysis of extracts from vehicle control or Nutlin-3a-treated (10 µM, 24 hr) C2C12 cells and showing increased reactivity with the p21 antibody. This recent study also shows specificity to p21 in Cre-treated Mdm2ec/+;HRASG12V and p53fl/fl;HRASG12V MEFs exposed to 1µM milademetan compared to controls (https://doi.org/10.1038/s41586-024-08318-8).

Antibody against puromycin was validated in our own study by including lysates from organoids that had been treated with cyclohexamide to block protein synthesis along with puromycin, and they were clear of any signal when subjected to western blot analysis and probed against puromycin (see Extended Data Figure 9m).

Beta-actin antibody specificity was validated with western blot analysis in a study that performed b-actin cell knockout experiments (https://doi.org/10.1016/j.plasmid.2018.08.005).

## Eukaryotic cell lines

Policy information about cell lines and Sex and Gender in Research

| | |
|---|---|
| Cell line source(s) | Organoid lines from murine intestinal crypts were generated in house at the Cancer Research UK Scotland Institute. Npm1fl/fl lines comprised of 2 female and 1 male lines. Apcfl/fl;KrasG12D/+ lines comprised of 2 male and 1 female lines. Apcfl/fl;KrasG12D/+;Npm1fl/fl lines comprised of 2 male and 1 female lines. |
| Authentication | None of the cell lines that were used were authenticated since they were primary organoid lines generated in house from biologically independent mice. |
| Mycoplasma contamination | Cell lines were not tested for mycoplasma contamination. |
| Commonly misidentified lines (See ICLAC register) | n/a |

## Animals and other research organisms

Policy information about studies involving animals; ARRIVE guidelines recommended for reporting animal research, and Sex and Gender in Research

| | |
|---|---|
| Laboratory animals | All experiments were conducted on mice that had been bred on a C57BL/6 background for at least three generations (n≥3) for homeostasis experiments, and at least four generations (n≥4) for CRC models. For intestinal experiments male and female mice were induced between 8-15 weeks of age or as soon as they reached 20 g of body weight. For hepatocyte specific genetic recombination of the Npm1fl/fl alleles, male and female mice between 2-4 months of age were induced with adeno-associated virus expressing Cre under the control of the thyroxine binding globulin (TBG) promoter (AAV8.TBG.Cre). |
| Wild animals | The study did not involve wild animals. |
| Reporting on sex | Sex was not considered in study design for all homeostasis experiments and CRC models. Short term experiments assessing proliferation in the liver tumour models were performed only in male mice due to large cell proliferation variability between the sexes. For the long term liver cancer studies both sexes were represented and analysed and presented independently. Sex of all mice used in each experiment is indicated in the source data of related panels. |
| Field-collected samples | The study did not involve samples collected from the field. |

| | |
|---|---|
| Ethics oversight | All animal experiments were performed in accordance with UK Home Office regulations (project licences 70/8646 and PP3907577), with the approval, and under the oversight, of the animal welfare and ethical review board (AWERB) of the University of Glasgow. All experiments performed adhered to Institutional guidelines in full. |

Note that full information on the approval of the study protocol must also be provided in the manuscript.

## Plants

| | |
|---|---|
| Seed stocks | *Report on the source of all seed stocks or other plant material used. If applicable, state the seed stock centre and catalogue number. If plant specimens were collected from the field, describe the collection location, date and sampling procedures.* |
| Novel plant genotypes | *Describe the methods by which all novel plant genotypes were produced. This includes those generated by transgenic approaches, gene editing, chemical/radiation-based mutagenesis and hybridization. For transgenic lines, describe the transformation method, the number of independent lines analyzed and the generation upon which experiments were performed. For gene-edited lines, describe the editor used, the endogenous sequence targeted for editing, the targeting guide RNA sequence (if applicable) and how the editor was applied.* |
| Authentication | *Describe any authentication procedures for each seed stock used or novel genotype generated. Describe any experiments used to assess the effect of a mutation and, where applicable, how potential secondary effects (e.g. second site T-DNA insertions, mosiacism, off-target gene editing) were examined.* |
