# Supplementary material for: Nucleophosmin supports WNT-driven hyperproliferation and tumor initiation
Source: Nat Genet. 2025 Dec 18;58(1):100–15. doi: 10.1038/s41588-025-02408-7 (PMC12807877; doi:10.1038/s41588-025-02408-7)

Extended Data fig. 9i - WB membranes

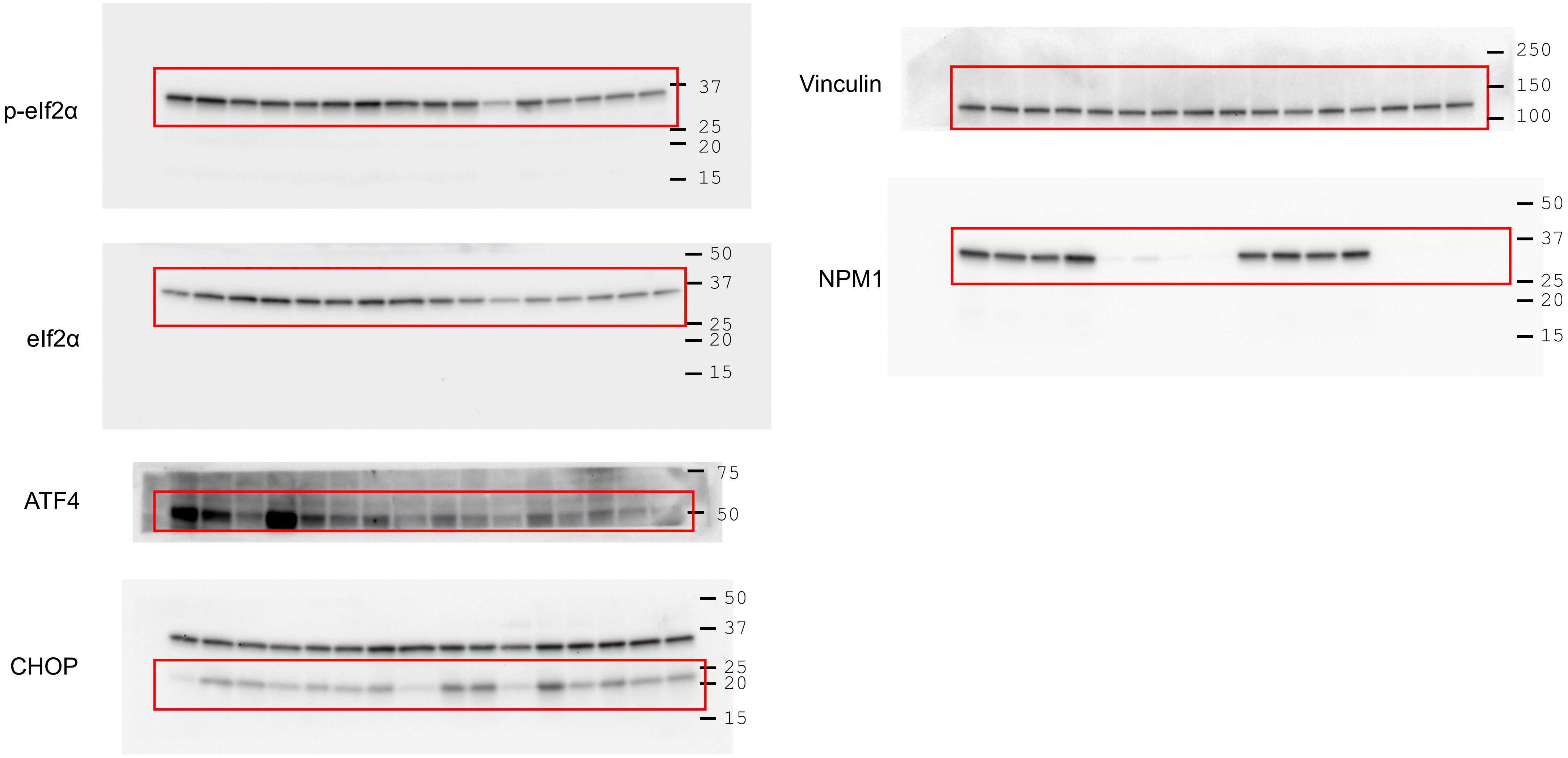

Extended Data fig. 9k - WB membranes

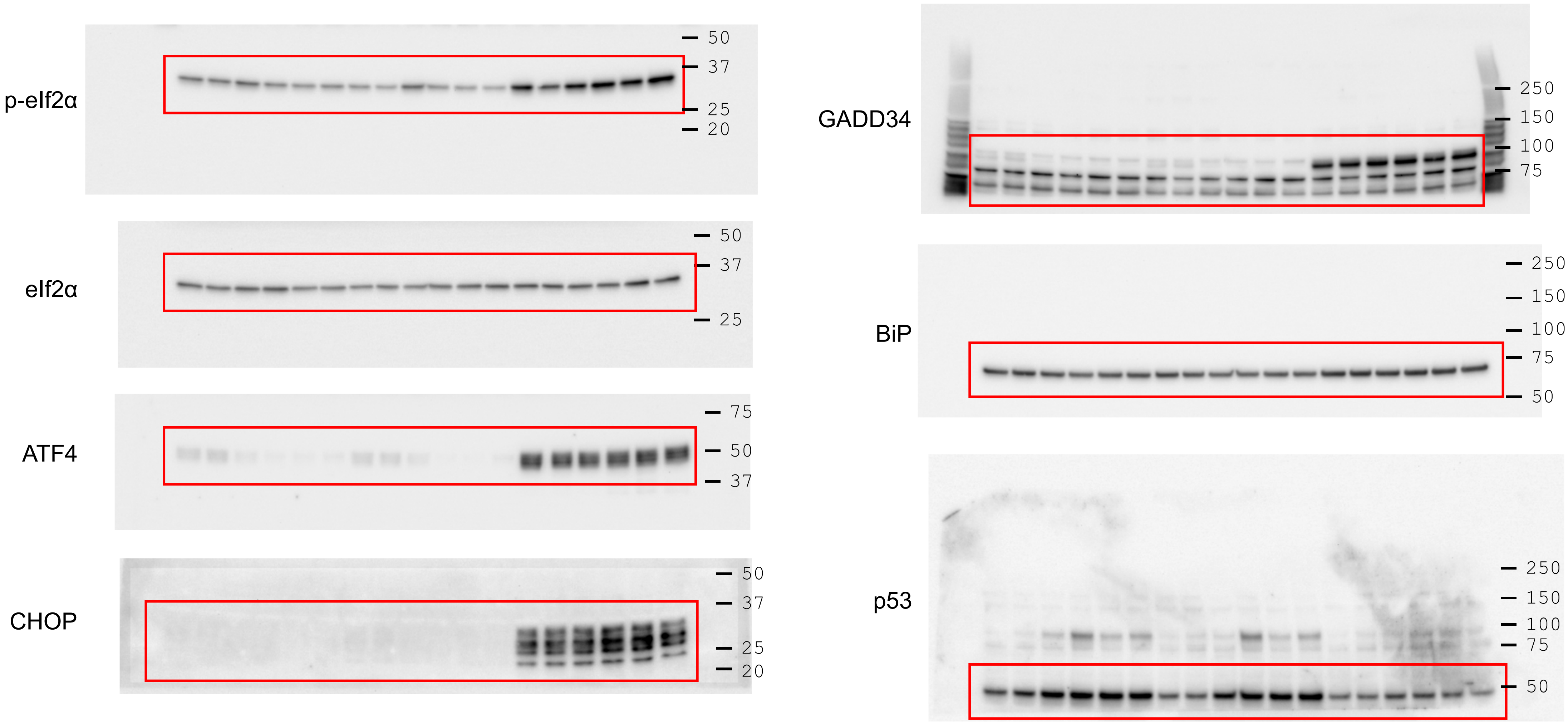

Extended Data fig. 9k - WB membranes

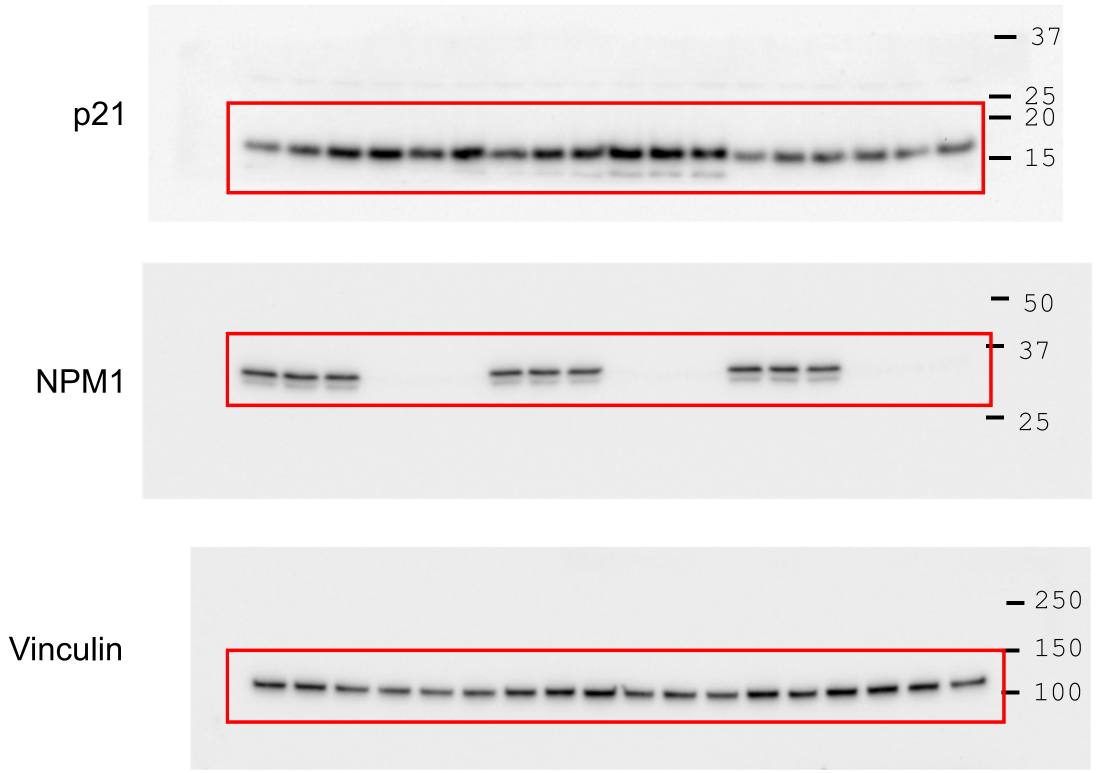

Extended Data fig. 9m - WB membranes

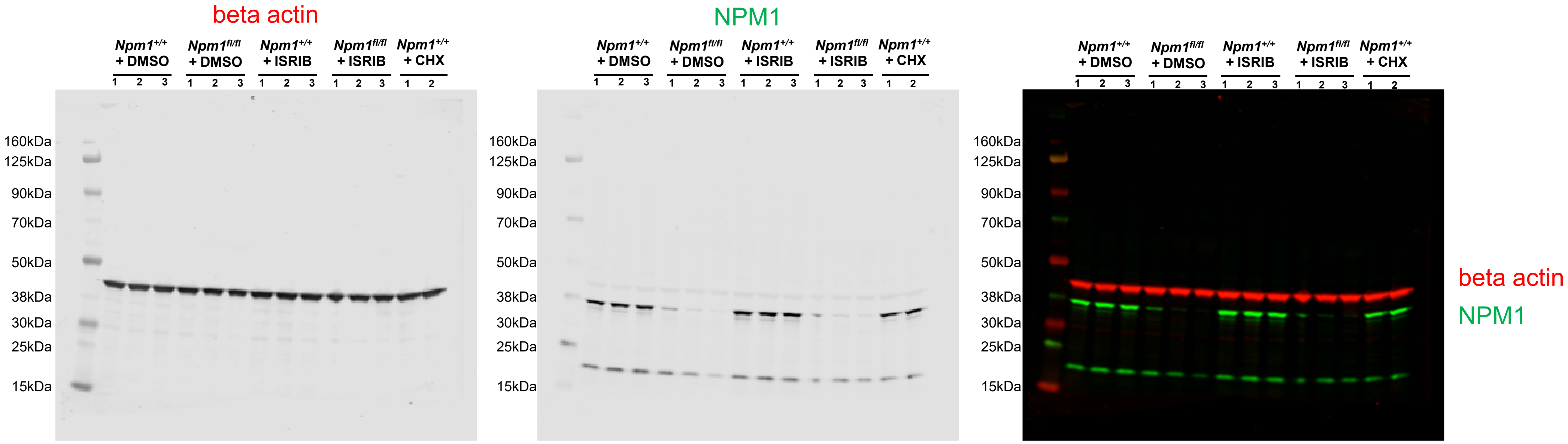

Supplement: Supplementary file 6 — Unprocessed immunoblots. [file 41588_2025_2408_MOESM6_ESM.pdf]
